# Supplementary material for: Study Protocol for Genomic Epidemiology Investigation of Intensive Care Unit Patient Colonization by Antimicrobial-Resistant ESKAPE Pathogens
Source: Methods Protoc. 2025 Dec 13;8(6):151. doi: 10.3390/mps8060151 (PMC12735888; doi:10.3390/mps8060151)
Supplement: Supplementary file 1 [file mps-08-00151-s001.zip › Table S1.pdf]

Antibiotic Susceptibility Testing Results

Facility: Moscow Multidisciplinary Clinical Center "Kommunarka"

Address: Moscow, Sosenskiy Stan str., 8c3

Laboratory: Microbiology Laboratory

Sample Information

**Analysis Number**	200 054 307	**Patient name**	
**Analysis Date**	09.07.2024	**Patient gender**	
**Department**	ICU 7	**Insurance policy #**	
**Medical Record №**	61492-21	**Clinician:**	
**Biomaterial**	Sputum		
**Sample Collection Date**	09.07.2024		

Bacterial Growth Detection

The following pathogens were isolated:

- *Acinetobacter baumannii* — 10^6 CFU/ml
- *Klebsiella pneumoniae* — 10^6 CFU/ml
- *Pseudomonas aeruginosa* — 10^6 CFU/ml

Antibiotic Susceptibility Profile

**Antibiotic**	*Acinetobacter baumannii*		*Klebsiella pneumoniae*		*Pseudomonas aeruginosa*	
	**S/R**	**MIC**	**S/R**	**MIC**	**S/R**	**MIC**
ESBL	—	—	Neg	—	—	—
Amikacin	R	—	R	≥64	S	≤2
Ampicillin	—	—	R	≥32	—	—
Gentamicin	R	—	R	4	—	—
Colistin	—	—	R	≥16	S	≤0.5

Meropenem	R	—	R	≥16	S	≤0.25
Trimethoprim/Sulfamethoxazole	R	—	R	≥320	—	—
Fosfomycin	—	—	R	64	—	—
Cefepime	R	—	R	≥32	I	2
Cefotaxime	—	—	R	≥64	—	—
Ceftazidime	—	—	R	≥64	I	2
Ciprofloxacin	R	—	R	≥4	I	≤0.25
Ertapenem	—	—	R	≥8	—	—
Imipenem	R	—	—	—	—	—
Levofloxacin	R	—	—	—	—	—

Legend

S/R: Sensitivity/Resistance classification

- **S:** Sensitive
- **R:** Resistant
- **I:** Intermediate resistance
- **MIC:** Minimum inhibitory concentration (µg/ml)
- **—:** Test not applicable or not performed for this species
- **Neg:** negative

Comments:

Date: ________________

Microbiologist Signature: ________________
